# Estimating Survival, Growth, and Morphometric Relationships of Blue Crab (*Callinectes sapidus*) in North Carolina Using Mark–Recapture and Bayesian Approaches

**DOI:** 10.3390/ani16152296

**Published:** 2026-07-24

**Authors:** Alex J. Rocco, Jie Cao

**Affiliations:** 1 Center for Marine Sciences and Technology, North Carolina State University, Morehead City, NC 28857, USA; jcao22@ncsu.edu; 2 Quinault Indian Nation, 1214 Aalis Dr, Taholah, WA 98857, USA

**Keywords:** mark–recapture, crustacean biology, growth, mortality, length–weight relationships, estuarine ecology, stock assessment, Cormack–Jolly–Seber modeling, Bayesian modeling, blue crab

## Abstract

Several of the North Carolina blue crab’s (*Callinectes sapidus*) life history parameters such as growth rate, natural mortality, and length–weight relationships would benefit from being updated or localized. To this end, we designed a mark–recapture study of blue crabs using coded wire tags to estimate carapace width (CW)-to-weight relationships, growth, and natural mortality in a North Carolina blue crab population. We used data from captured blue crabs to estimate their CW-to-weight relationships and used recapture data to estimate growth rates via the Fabens method using both frequentist and Bayesian approaches. Additionally, we built a Cormack–Jolly–Seber (CJS) model to estimate apparent survival. Our CW-to-weight estimates fell largely between the CW-to-weight estimates from the Chesapeake Bay and Florida stocks, suggesting possible regional variation. Our frequentist and Bayesian growth rate estimates had higher k values than other estimates based on a limited number of growing recaptures. There was no single CJS model that was strongly supported by data, but we identified important covariates that inform the precision and accuracy of the CJS model for future studies. Our results suggest that coded wire tags can be useful for mark–recapture studies of species without permanent hard parts, but considerable effort may be necessary to produce viable results.

## Simple Summary

The North Carolina blue crab’s (*Callinectes sapidus*) growth, natural mortality, and length–weight relationship would benefit from being updated or localized. To do this, we designed a mark–recapture study of blue crabs to estimate these parameters. We used data from captured blue crabs to estimate their length–weight relationships and growth rates and built a model to estimate their apparent survival using this data. Our length–weight estimates fell largely between the length–weight estimates from Chesapeake Bay and Florida, suggesting possible regional variation. Our growth data was limited but nonetheless provided useful estimates of growth rates. Our model did estimate apparent survival but could not find a strongly correlated combination of groups and covariates. However, we did find some covariates that would likely inform future studies. The results of this study suggest that mark–recapture studies of animals without permanent hard parts are feasible, but considerable effort is necessary to produce viable results.

## 1. Introduction

Mark–recapture studies have long been a useful tool for ecologists to better understand the dynamics of animal populations. Marking of individuals is conducted using a variety of different methods spanning a wide range of complexity [1,2]. Mathematical and statistical models are often used to analyze the data produced from these mark–recapture studies to estimate life history parameters [3,4]. Mark–recapture studies are especially important in fisheries because of the difficulty or impossibility of directly observing population demographic parameters in aquatic populations such as abundance and mortality [5]. These parameters are central to stock assessments and management of commercially and recreationally important fish and shellfish species [4,6,7].

Blue crabs (*Callinectes sapidus*) range from the Maritime Provinces in Canada to Caribbean Brazil and support valuable commercial and recreational fisheries throughout much of the western Atlantic and Gulf of Mexico [8,9]. In North Carolina, blue crab fishery is the most commercially valuable fishery in the state, averaging 28.0 million dollars per year from 2004 to 2016 [9]. Stock assessments that support this fishery rely on biological parameters such as growth, mortality, and morphometric relationships to inform regulations and ensure the sustainability of the fishery. The stock was listed as overfished, with overfishing occurring in the most recent stock assessment by the North Carolina Division of Marine Fisheries [9]. However, some of the parameter values used in recent assessments have been derived from older studies or borrowed from other regions. Specifically, the 2018 stock assessment sourced their carapace width (CW)-to-weight equations from stock assessments from Chesapeake Bay and Florida [9]. Additionally, estimates of growth are sourced from the 2004 North Carolina stock assessment as well as estimates from Delaware, Florida, and Chesapeake Bay. Use of life history parameters from outside of North Carolina run the risk of misrepresenting critical life history information about the stock, which could affect the accuracy and precision of blue crab stock assessments in North Carolina. Although these sources provided the best available information at the time, changing environmental conditions, habitat dynamics, and fishery pressures create a need to update key life history parameters using contemporary, region-specific data to ensure that future stock assessments accurately reflect the present state of the North Carolina blue crab population.

Estimating growth in blue crabs and crustaceans in general is challenging because crustaceans grow discontinuously through molts. In blue crabs, growth rates have been estimated using the continuous von Bertalanffy growth function (VBGF) [8], and it was found that these growth rates, while not physiologically accurate, produced similar results to models that more closely simulate growth in crustaceans and are viable in modeling frameworks that describe growth at the population level [8]. Even so, obtaining data to inform the VBGF parameter estimates can be difficult. Individuals captured in mark–recapture studies may show no visible size change between capture events if molting has not occurred [8,9]. In addition, crustaceans generally lack permanent hard parts that can be used reliably for aging in the same way as calcified structures in fishes. For these reasons, field-based estimates of age and growth are often uncertain. However, size increment data collected from mark–recapture experiments provide a practical alternative for estimating growth parameters [4].

Survival-related parameters are similarly difficult to estimate. Natural mortality (M) is one of the most influential parameters in fisheries stock assessments, as it directly affects estimates of population productivity, exploitation rates, and reference points [10]. However, estimates of M are inherently uncertain and can vary substantially across space, time, and life history stages due to differences in environmental conditions, predation, and individual physiology [10,11]. In the case of the North Carolina blue crab, assessments have historically relied on estimates derived from Hoenig’s (1983) empirical relationship between M and maximum observed age, as applied in the 2004 stock assessment [8,12]. While this approach provided a practical foundation, it assumes that mortality remains constant across years and regions—an assumption that may not hold under changing ecological and fishery conditions. Mark–recapture approaches can help address this uncertainty by providing empirical estimates of natural mortality or apparent survival. Apparent survival differs from true survival because apparent survival does not differentiate between mortality and emigration from the system [13,14]. While measures of apparent survival are generally less precise, apparent survival estimates can provide a valuable benchmark for evaluating mortality-related assumptions used in assessment models. Improving mortality estimates for the North Carolina blue crab stock is important to recovering the stock from its overfished and overfishing status [9].

The aim of this study was to estimate key life history parameters—including natural mortality, growth, and carapace width (CW)–to–weight relationships—for the North Carolina blue crab population. To achieve this, we conducted a mark–recapture study from 2022 to 2024 using Northwest Marine Technology, Inc. (Anacortes, WA, USA) injectable coded wire tags (CWTs) at three sites within the Newport River estuary in eastern North Carolina. Following field collection and processing of tagged individuals, we estimated survival using a Cormack–Jolly–Seber (CJS) model, growth parameters using both Bayesian and frequentist reparameterizations of the von Bertalanffy Growth Function (VBGF), and CW–to–weight relationships using Ricker’s (1975) allometric equation [4,15,16,17,18]. Together, these analyses provide updated, region-specific estimates of critical life history parameters for the North Carolina blue crab stock, supplying more current and biologically realistic inputs for future stock assessments and management decisions.

## 2. Materials and Methods

### 2.1. Study Sites

**Figure 1:**
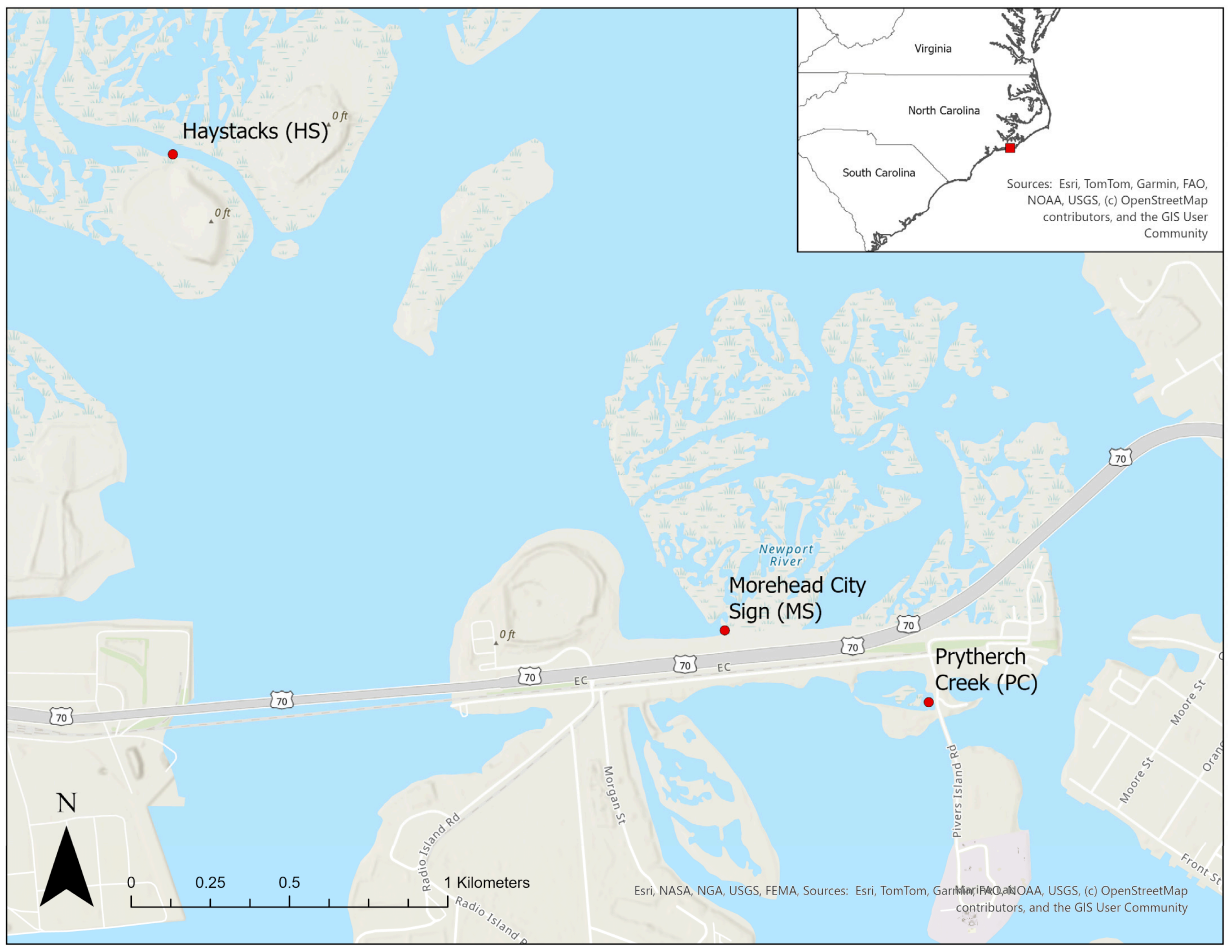
Map of the sampling sites in the Newport River estuary, North Carolina, USA.

Field sampling was conducted in the Newport River estuary, North Carolina, USA, at three salt-marsh sites: Haystacks (HS; 34°44′01.2″ N, 76°41′42.3″ W), Prytherch Creek (PC; 34°43′17.5″ N, 76°40′27.9″ W), and Morehead City Sign (MS; 34°43′22.3″ N, 76°40′47.0″ W) (Figure 1). The initial two sites, HS and PC, were selected based on Johnson and Eggleston (2010), and MS was added in 2024 to improve catch rates [19]. All three sites are in the Newport River Estuary but differ in marsh structure, accessibility, and degree of openness to the main estuarine channel.

HS is a relatively large and open salt marsh with many tidal creeks crisscrossing throughout its range, and is only accessible by boat. The substrate at HS was characterized by sand and oyster bars. While depth is highly variable in the system, much of the estuarine bottom at our sampling site was permanently submerged. HS was generally sampled around high tide to ensure boat access to the site. PC is an enclosed area of salt marsh with a single direct connection to the Newport River; during sampling, it was accessed both by boat and by wading. The substrate at PC was mostly mud with oysters scattered throughout. The wadable parts of this site would often not be submerged at low tide, while the parts accessible by boat would remain submerged. PC was sampled both at low tide and high tide depending on whether we accessed the site by wading or boat, respectively. MS is a medium-sized salt marsh with some tidal creeks throughout. The salt marsh opens to the Newport River except on its southern edge, where it is enclosed by the shoreline of Radio Island. During sampling, this site was accessed by wading at low tide. The substrate at MS was mostly mud with fairly large oyster bars. The main creek channels at MS are permanently submerged, yet many shallower portions, particularly around the creek edges, would become exposed at low tide. At HS and PC, sampling took place from April through November, 2022–2024. During the 2024 season, we were only able to sample HS once because of technical difficulties with the boat we were using. At MS, sampling took place from May to August 2024.

### 2.2. Sampling Procedure

We sampled crabs across all sites a total of 55 times from 2022 to 2024, with 11 sampling events in 2022, 26 sampling events in 2023, and 18 sampling events in 2024. Sampling was designed to maximize capture rates of blue crab large enough to tag (>22 mm CW) across a size range. As such, we used a combination of active and passive gear types across all sites, and sampling methods were adjusted over time to improve catch rates. Active sampling consisted of a 2 m beam trawl (0.76 cm mesh, 0.38 cm mesh cod end) and opportunistic hand capture during other sampling activities. Passive sampling consisted of five trap types spanning a broad size selectivity range: peeler pots (58 cm L × 58 cm W × 48 cm H, 3.5 cm mesh), Promar^®^ (Gardena, CA, USA) standard crab pots with escapement devices blocked by 0.127 cm window screen mesh (61 cm L × 61 cm W × 28 cm H, 5.08 cm mesh), Eagle Claw^®^ (Denver, CO, USA) cylindrical minnow traps (42 cm L × 19 cm diameter, 1 cm mesh), and two Protoco^®^ (North Plains, OR, USA) rectangular minnow traps (46 cm L × 28 cm W × 14 cm H and 61 cm L × 23 cm W × 23 cm H, 2.54 cm mesh). Escapement devices on the standard pots were blocked to widen the selectivity range of the trap. Traps were baited with cut baitfish. Initially, our soak times were roughly 24 h, coinciding with tidal cycles. As our sampling methods evolved, the soak times were shortened as it was found that more crabs were caught with soak times of 4 h or less. In total, we captured and tagged 364 individuals at PC, 128 individuals at HS, and 96 individuals at MS.

### 2.3. CW-to-Width Parameter Estimation

Sex-specific CW-weight relationships were estimated using data from both initial captures and recaptures. Before analysis, sponge females and any individuals recorded as mutilated or missing limbs were excluded. Males and females were analyzed separately because of morphological differences between the two [20]. The final dataset used for CW-weight estimation included 345 males and 224 females.

We modeled the relationship between CW and body mass using the allometric equation:

(1) $$W=\alpha *{CW}^{\beta }$$

where *W* is body weight and *CW* is the carapace width.

### 2.4. Growth Modeling

Growth analyses were based on recaptured individuals that showed measurable growth between capture and recapture events. We first estimated growth using the reparameterized von Bertalanffy growth function described by Fabens 1965 [21,22]:

(2) $$L_{T2}-L_{T1}=∆L=\left(L_{\infty }-L_{T1}\right)(1-e^{-K∆T})$$

where *L_t_* represents the length at age *t*, *L*_∞_ is the asymptotic maximum length, *k* represents the Brody coefficient describing the steepness of the curve, and capture and recapture events are represented by *T*1 and *T*2, respectively, with the condition that *T*1 < *T*2.

We then fit a Bayesian Fabens method (BFa) using narrow (BFaI) priors to compare with the frequentist estimates. Prior distributions for each parameter were specified as follows [4]:

(3) $$∆L_{i}=L_{\infty }-\left(L_{\infty }-L_{T1,i}\right)e^{\left(-k∆T_{i}\right)}+\varepsilon_{i}\;\varepsilon_{i}\sim \;iid\;N\left(0,\;\sigma^{2}\right)\;\;L_{\infty }\sim N\left(u_{\infty },\;\sigma_{\infty }^{2}\right)\;\;k\;\sim \;U\left[lb.k,\;ub.k\right]\;\;\sigma \;\sim \;U\left[lb.\sigma ,\;ub.\sigma \right]\;$$

where *σ* is the error term’s standard deviation, and *μ*_∞_, *σ^2^*_∞_, *lb.k*, *ub.k*, *lb.σ*, and *ub.σ* are hyperparameters sourced using prior VBGF models of blue crab growth. The hyperparameter values *u*_∞_ and *σ*_∞_ were set to 206.9 and 43, respectively; these values were set somewhat arbitrarily based on blue crabs’ general size range. For *K*, hyperparameters *lb.k* and *ub.k* were set as 10^−10^ and 100, respectively; and for *σ*, *lb.σ* and *ub.σ* were set as 10^−10^ and 10^3^, respectively. Starting points for *L*_∞_ and *k* were calculated as follows:

(4) $$init.L_{\infty }=L_{max}/0.99\;init.k=med(\frac{-\ln\left(\frac{{(init.L}_{\infty }-L_{T2,\;i})}{\left({init.L}_{\infty }-L_{T1,i}\right)}\right)}{{∆T}_{i}})$$

Using this framework, *L*_∞_, *k*, and *σ* are not fixed, but instead are effectively random variables with fixed hyperparameters. We ran the BFa model with 3 Markov chains, with 10,000 iterations and 5000 iterations discarded after a burn-in period. Once both the frequentist and Bayesian VBGF parameters were estimated, we estimated length-at-age data using an equation from Dureuil et al. 2022 [4]:

(5) $$t=\frac{{log}_{e}\frac{\left(L_{\infty }-L_{0}\right)}{\left(L_{\infty }-L\right)}}{k}$$

where *t* represents age, *L*_0_ is the length at birth, and *L* is the given length of an individual. *L*_0_ values for our models were calculated using the VBGF, with *t* set to 0, since we are estimating CW at age 0:

(6) $$L_{t}=L_{\infty }(1-e^{\left(-k\left(t-T_{0}\right)\right)})$$

where *t* is age, and *T*_0_ is the age at length 0; *T*_0_ was set as 0, since we did not have an estimate of *T*_0_. The calculation of these growth curves allowed for easy comparison between our Bayesian and frequentist parameter estimates, as well as comparison of these values to past parameter estimates and estimates from other regions, such as Chesapeake Bay.

### 2.5. Cormack–Jolly–Seber Modeling

We used CJS models to estimate apparent survival (*ϕ*) and recapture probability (*p*) from the mark–recapture data. Models were fit in R using the Rmark package in conjunction with the MARK program [21,22,23,24]:

(7) $$\left[\begin{array}{c} \phi \\ p \end{array}\right]={\left[1+exp(-X\beta_{i})\right]}^{-1}$$

where *X* is the design matrix, and *β_i_* is the vector of parameters for either *p* or ϕ.

Capture histories (CH) were constructed for each tagged crab using a sequence of zeros and ones, where 1 indicated capture during a sampling occasion and 0 indicated non-detection. Since we did not recapture any individual at a site different than their initial capture site, we constructed separate datasets for HS, PC, and MS, as well as one combined dataset containing all sites. Because preliminary results suggested that changes in sampling method between 2022 and later years may have influenced the PC results, we analyzed PC in two ways: one with data from 2022 to 2024, and one with only the data from 2023 and 2024.

We considered biologically plausible effects of sex, year, trap, month, site and CW on *ϕ* and *p*. In the site-specific datasets, candidate grouping variables were sex, year, trap and month; CW was included as a continuous variable. In the combined dataset, site was added as an additional grouping factor. We first fit a null model with no covariates or grouping effects and then screened single-factor and single-covariate models. Variables that consistently produced convergence issues and unstable estimates were removed from subsequent candidate models.

We then fit candidate models containing supported combinations of grouping factors and covariates for *ϕ* and *p*. Real parameters were estimated using the default inverse-logit link in RMark. Candidate models were compared using Akaike’s information Criterion (AIC) corrected for small sample size. This model-selection workflow was repeated for five datasets: PC including 2022, PC excluding 2022, HS, MS and the full combined dataset.

## 3. Results

### 3.1. CW-Weight Relationships

Sex-specific CW-weight relationships differed between males and females. The estimated allometric relationships for each sex were as follows:

(8) $$Weight\;(M)=0.000723*{CW}^{2.534967}\;Weight\;(F)=0.006413*{CW}^{2.032393}$$

**Figure 2:**
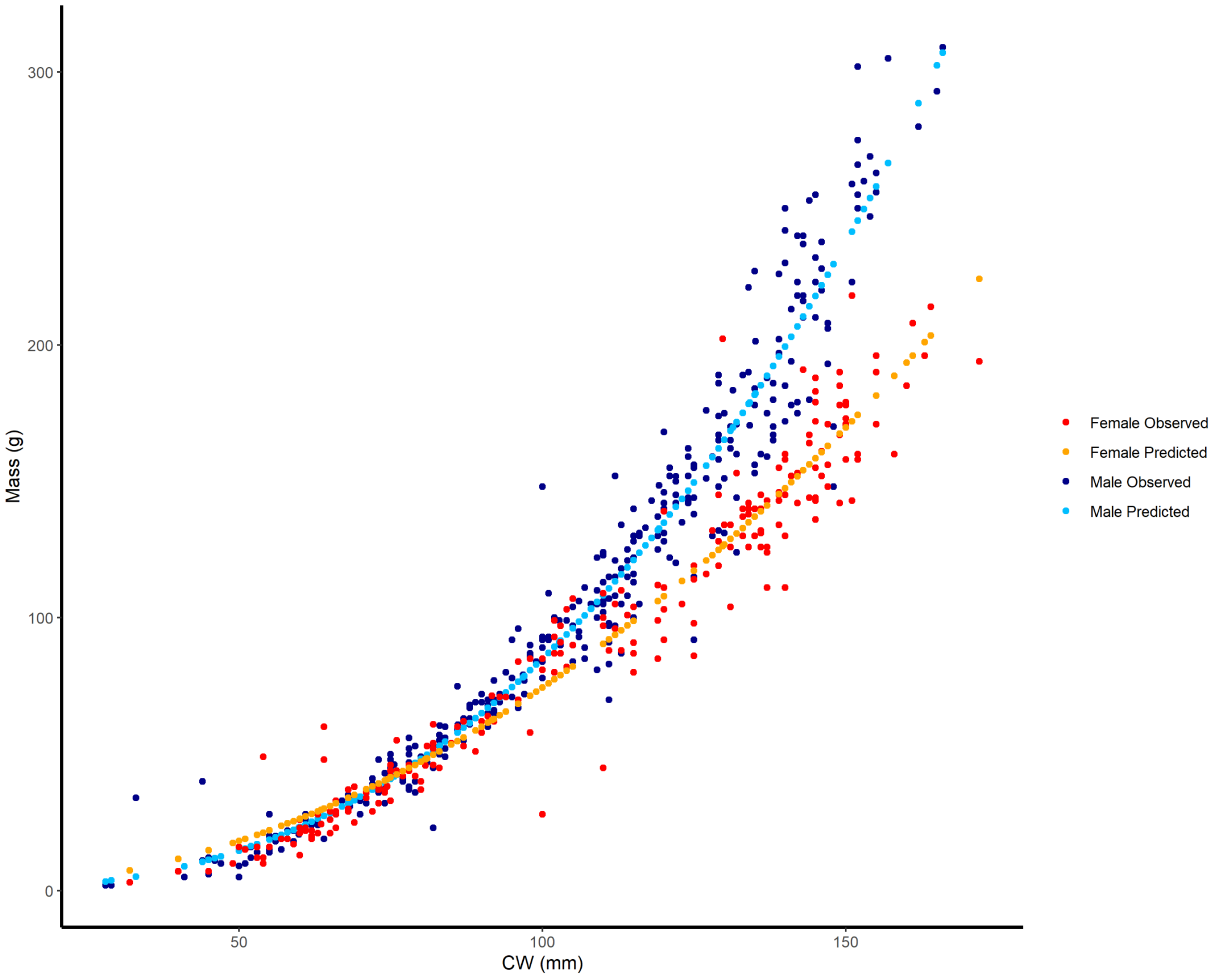
Calculated predictions of male and female CW-to-weight ratios plotted against the observed values for each sex.

At smaller carapace widths, predicted male and female masses were similar; however, the relationships diverged with increasing CW. When CW was ~75 mm, males generally began to exceed females in predicted masses. This separation became more pronounced at larger body sizes (Figure 2).

**Figure 3:**
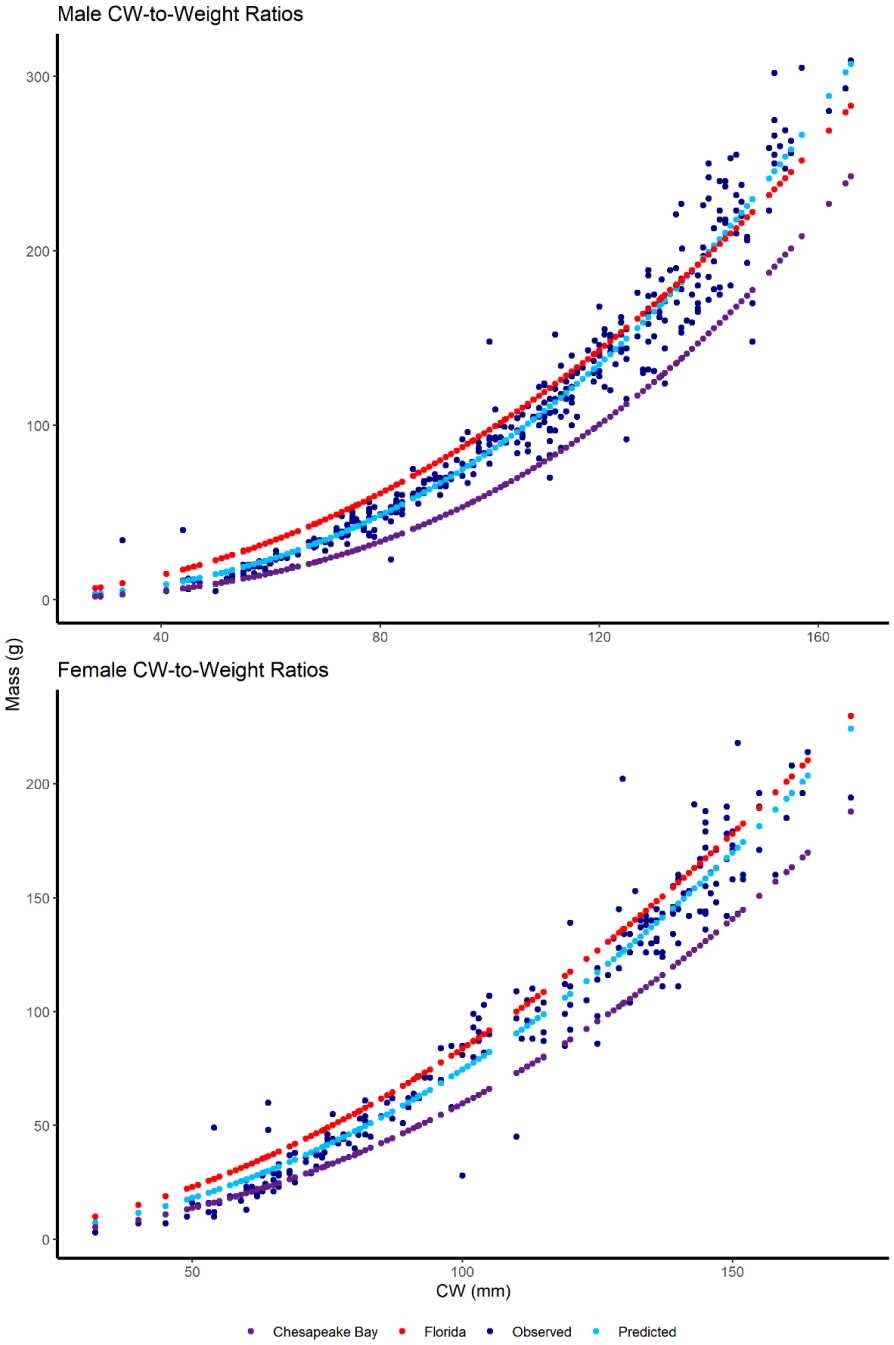
Comparison of observed and predicted CW-to-weight ratios using our parameter estimates, estimates from Florida [26], and estimates from Chesapeake Bay [27].

When compared with published CW-weight relationships from Chesapeake Bay and Florida, the North Carolina male and female estimates generally fell between the corresponding regional curves across most of the observed size range (Figure 3) [25,26]. Florida estimates showed largely higher weights at all CW estimates except for males > 130 mm CW (Figure 3) [26]. No significant differences between our CW-weight relationships and the Florida CW-weight relationships were found for either sex (*t*_2688_ = −1.3841, *p* = 0.1668 and *t*_2446_ = −1.5744, *p* = 0.1161 for males and females, respectively). Significant differences were found between our data and the Chesapeake Bay data for both sexes, however (*t*_2688_ = 5.9082, *p* = 5.44 × 10^−9^ and *t*_2446_ = 3.6664, *p* = 0.0002757 for males and females, respectively).

### 3.2. Growth

**Figure 4:**
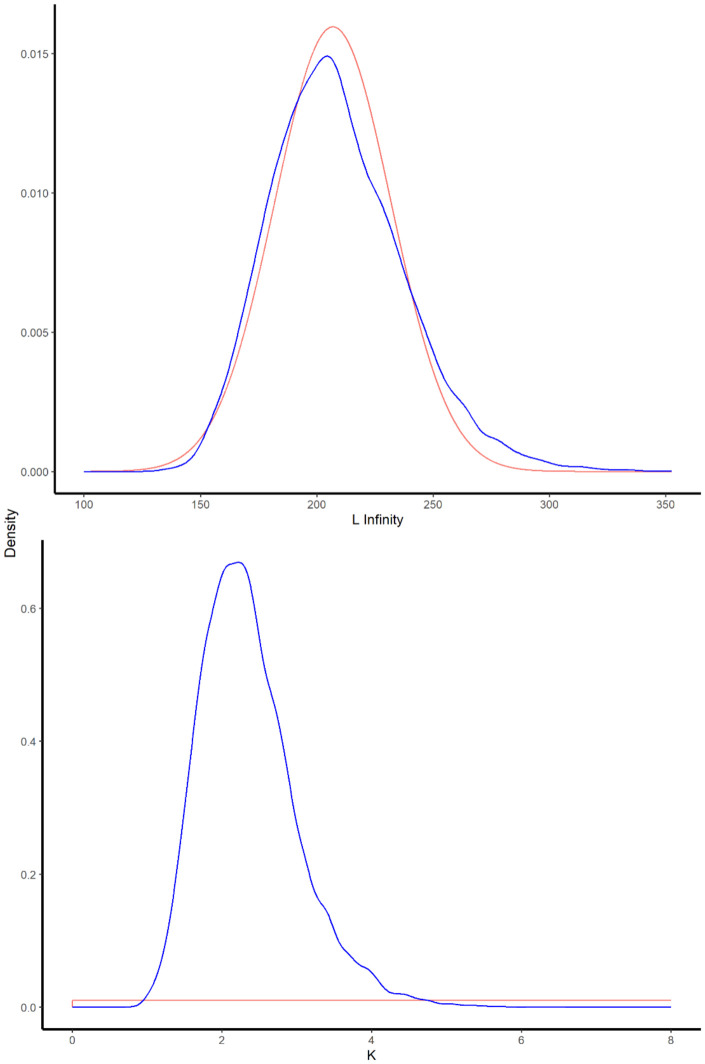
Prior (red) vs. posterior (blue) distributions of L_∞_ and K for our BFa model. The prior distribution for L_∞_ is represented by a normal distribution with a mean of 206.9 and a 99% quantile of 280. The prior distribution for K was a uniform distribution with a lower bound of 10^−10^ and an upper bound of 100.

Posterior estimates produced by the BFa model converged upon an L_∞_ of 205.24 (R-Hat = 1.00, ESS = 4525) and a K of 2.28 (R-Hat = 1.00, ESS = 4421). The 95% confidence intervals for L_∞_ were between 160.89 and 271.69, while confidence intervals for K were between 1.37 and 3.89 (Figure 4 and Table 1). Posterior distributions for L_∞_ and K resembled right-skewed normal distributions centered on their respective estimate (Figure 4). The L_∞_ estimate produced by our frequentist Fabens model was 235.32, and the K estimate produced by our frequentist Fabens method was 1.83. Both of these estimates fell within the 95% confidence interval of the BFa model, suggesting that the Bayesian estimate can produce similar results to the frequentist estimate. When both estimates were compared to Eggleston and Johnson 2004’s estimates of VBGF parameters from size-at-age values for the North Carolina blue crab, the Eggleston and Johnson 2004 estimate of L_∞_ (216.9) fell within the confidence interval of our BFa analysis, but its estimate of K (0.47) did not fall within the confidence interval [8].

**Figure 5:**
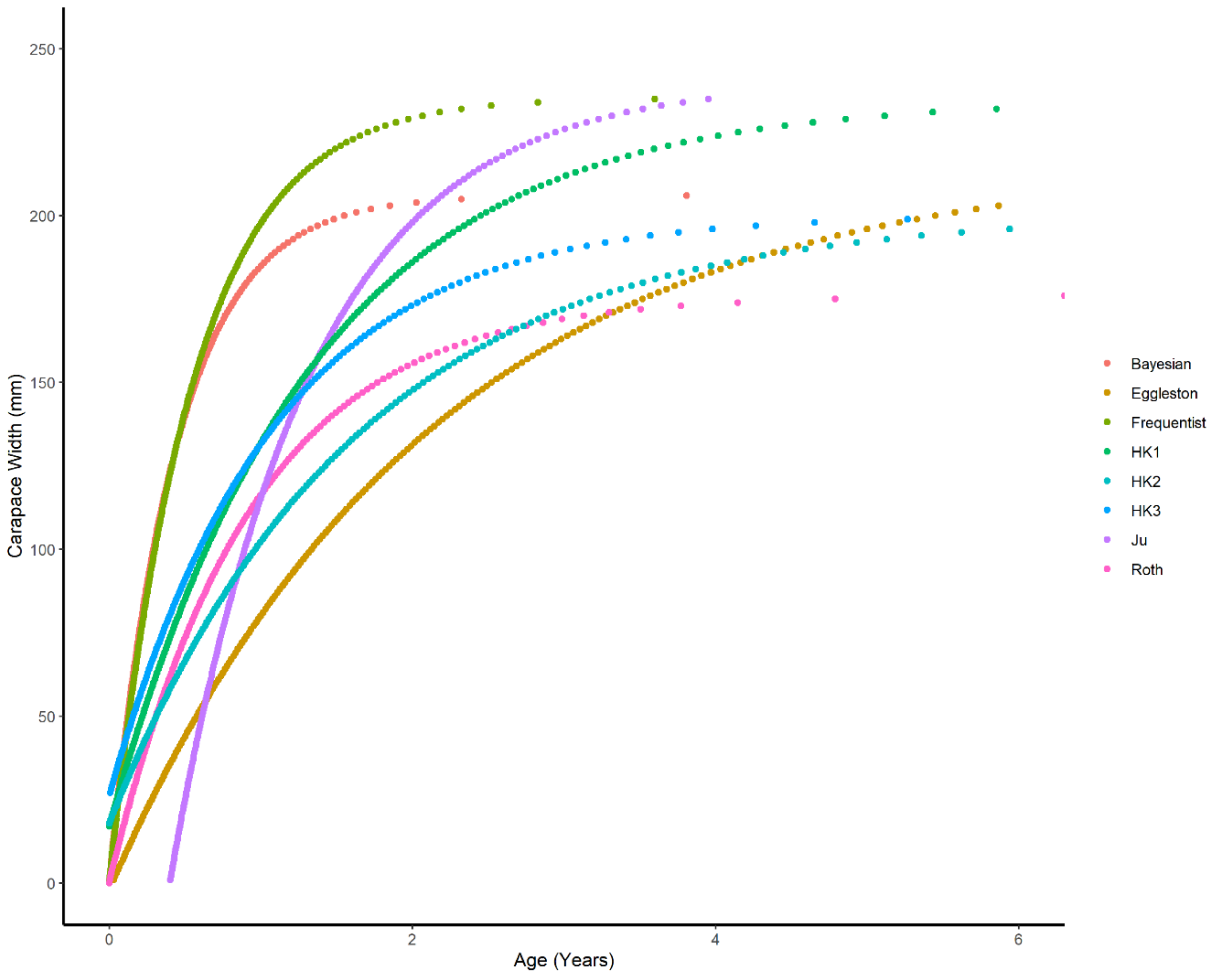
Comparison of growth curves using our parameter estimates with curves from other studies (Table 1).

When our BFa estimates are compared to VBGF parameter estimates for Chesapeake Bay and Delaware Bay, all L_∞_ estimates fell within the confidence intervals estimated by the BFa model, and none of the K values fell within the respective BFa confidence intervals (Table 1). This suggests that the BFa estimate of L_∞_ may be reliable, but the BFa estimate of K may not be an entirely accurate estimate. When the VBGF parameters for all these models are converted into a growth curve using Equation (5), the asymptotes for the BFa and Frequentist Fabens L_∞_ appear similar to the other curves, but the slopes of our two models are considerably steeper than any of the other models, suggesting that the model estimates based on our data may produce unrealistic growth rates if used to construct a growth curve (Figure 5).

### 3.3. CJS Model Comparison

In total, we ran 32 models each for PC with and without 2022, 14 models for HS, 15 models for MS, and 42 models for the combined dataset (Supplementary Tables S1–S5). The differences between the number of models between the five datasets is largely due to the differences between viable groups and models; depending on the data, some of the groups that worked for other datasets would not work for a given dataset. The addition of the Site group for the combined dataset also increased the number of models we tested. AICc scores for each model generally stayed within 10 points of each other. Models with AICc scores more than 10 points greater than the minimum AICc in this dataset were removed from analysis. For PC with and without 2022, the lowest AICc (AICc = 302.79 and 277.52, respectively) was produced by the model with year as a group for both *p* and ϕ (Supplementary Tables S2 and S3). For HS, the null model had the lowest score (AICc = 22.39) (Supplementary Table S4). For MS, a model with no groups for ϕ and trap as a group for *p* had the lowest AICc (AICc = 62.92) (Supplementary Table S5). The combined dataset model with the lowest AICc was the model with CW as a covariate for ϕ and no groups or covariates for *p* (AICc = 428.02) (Supplementary Table S1). Because of the relatively close AICc scores between different models in all datasets, we will instead focus our results on the effects of each group and covariate, and any potential interactions between them.

CW was used as a covariate for models in all datasets: CW’s coefficient was mostly negative, apart from two model configurations in PC. For models that incorporated both year and CW in *p* and ϕ, CW’s coefficient was positive. When isolated into three separate datasets, the years 2022, 2023, and 2024 all have a negative CW coefficient. Models grouped by year had marked differences in values of ϕ and *p* between each group. Models with year as a group often had highly variable ϕ values, marked by very low survival in year 2022, low survival in 2023, and moderate to high survival in 2024 (Supplementary Table S2). ϕ values for models without year as a group largely fell between the yearly estimates in year-grouped models. When models were grouped by sex, ϕ values were generally lower in males than females. Likewise, *p* was generally lower for males than females when sex was a grouping, but its coefficient was positive in a few site-grouped models in the complete dataset.

Because the rest of our datasets were site-specific, we only used site in our complete dataset to see how the precision of our estimates changed when estimating *p* and ϕ compared to the sites measured separately. Like year, grouping our complete datasets by site produced highly variable values of ϕ (Supplementary Table S1). Compared to the separate datasets, the inclusion of site as a group for the complete dataset seems to produce less extreme results compared to the most extreme values produced by the individual datasets. The relationship between Site and *p* was less clear; the sign of the coefficient for both MS and PC did not show a specific positive or negative trend.

**Figure 6:**
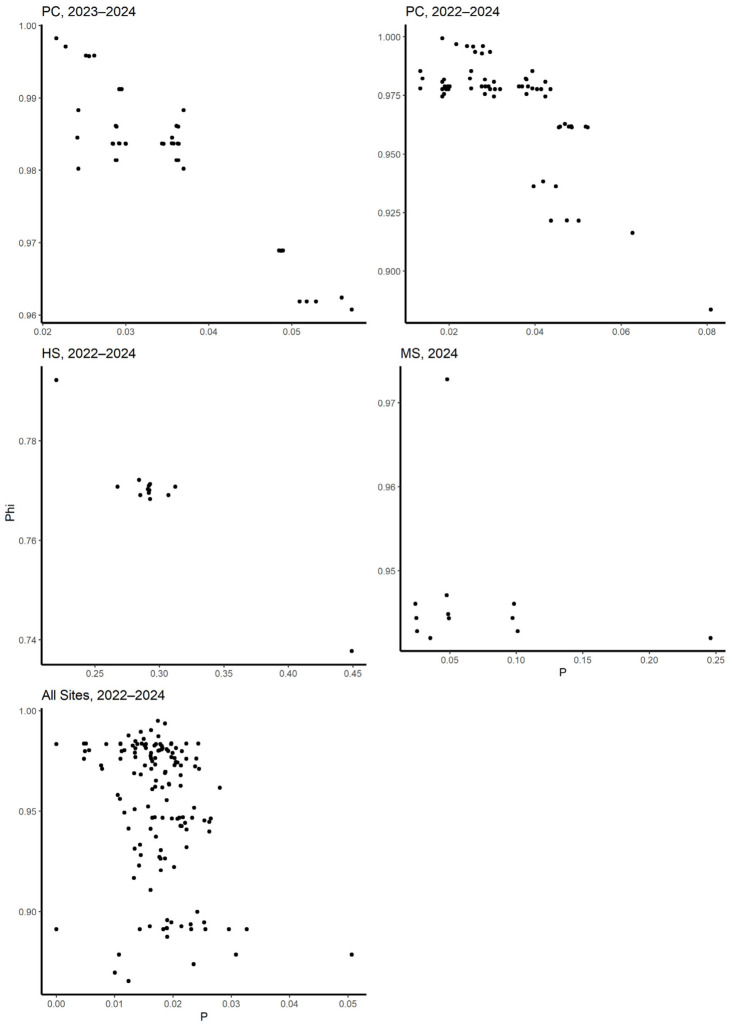
Relationships between estimated values for *p* and ϕ in each dataset.

**Figure 7:**
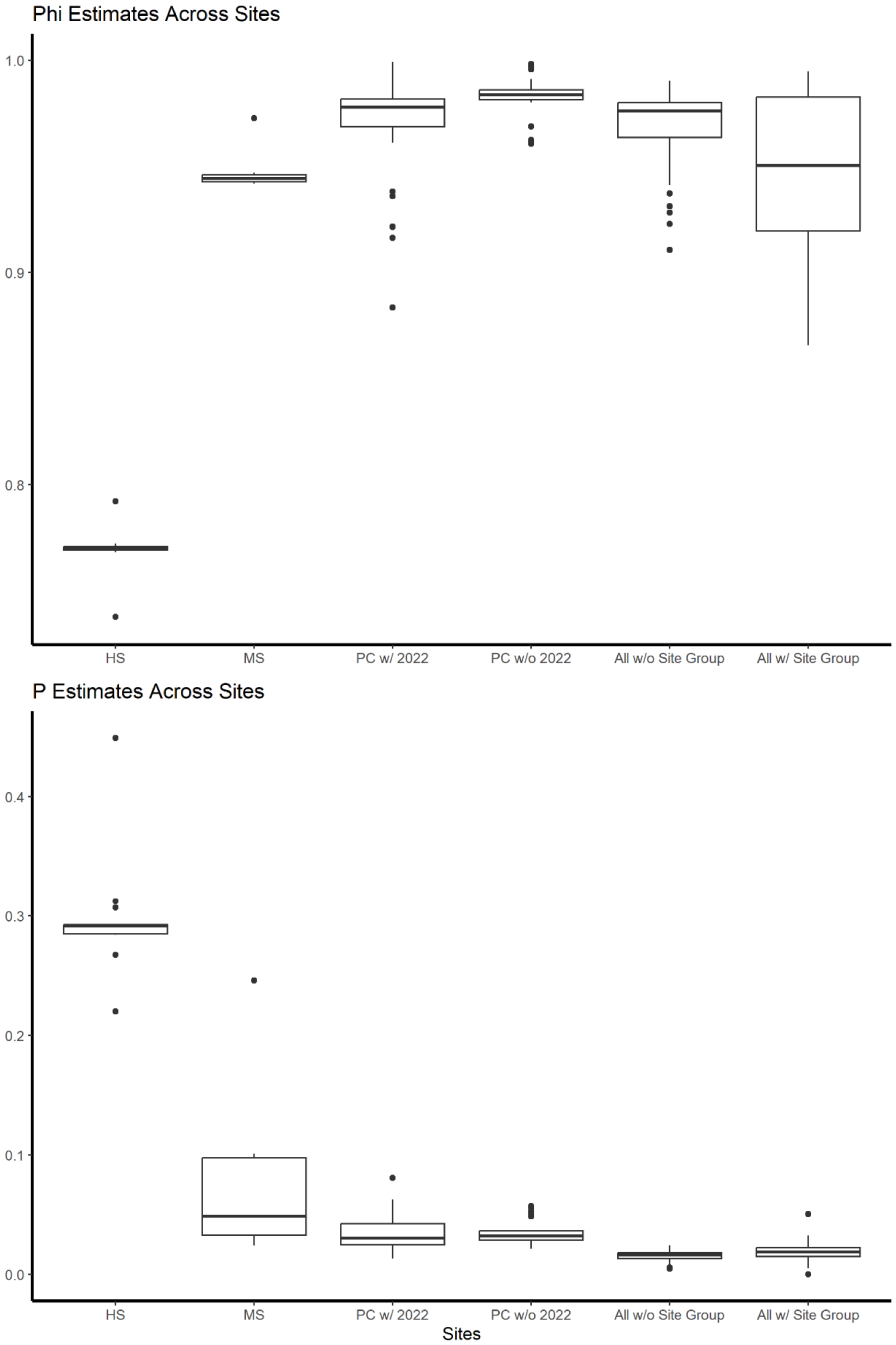
Estimates of ϕ and *p* across sites; for PC and the combined dataset, estimates are provided both with and without 2022.

When *p* and ϕ are plotted, there is seemingly a negative correlation between the two parameters in both the PC dataset with 2022 and without 2022 (Figure 6). The relationship between *p* and ϕ is less clear in MS, HS, and the combined dataset. When estimates of *p* are compared between each site group, the HS *p* estimate is generally much higher than the other sites’ estimates. MS’s estimate is relatively similar to the other sites, but it is considerably less precise (Figure 7). PC with 2022’s estimate is also somewhat less precise; PC without 2022 and all sites combined with and without site groups had similar estimates and precision for *p*, with all sites combined without a site group having the highest precision (Figure 7). Conversely, the HS estimate for ϕ is considerably lower than the other estimates (Figure 7). MS’s ϕ estimate is lower than the other sites and is much more precise than the MS *p* estimate (Figure 7). Both PC ϕ estimates and the all-sites-combined dataset without site groups estimated similar values for ϕ, but PC without 2022 was considerably more precise than the other two sites (Figure 7). The complete dataset with site groups provided the least precise estimates of ϕ, with its upper and lower quantiles overlapping with MS, all without site group, and PC with and without 2022 (Figure 7). There were also generally more outliers in estimates of ϕ than in estimates of *p*, suggesting that estimation of ϕ was generally less precise than estimation of *p* (Figure 7).

## 4. Discussion

This study used coded wire tag mark–recapture data to estimate sex-specific CW-weight relationships, evaluated growth using Fabens-based von Bertalanffy approaches, and estimated apparent survival using CJS models for blue crab in the Newport River estuary, North Carolina. Overall, the study provides updated, region-specific biological information for North Carolina blue crab and shows that coded wire tagging experiments can be useful for demographic inference in crustaceans, although inference on growth and survival was limited by sparse recaptures, seasonal sampling, site openness, and variation in field methods among years.

The difference in CW-to-weight relationships between male and female blue crabs and other Brachyurans is well-documented [20,30,31]. Due to developmental and morphological differences between male and female blue crabs, it is standard practice to develop separate estimates of CW-to-weight relationships for each sex [9,25,26].

The equations of CW-to-weight that we calculated for males and females based on our observed carapace widths and weights largely fell between the CW-to-weight equations in Chesapeake Bay and Florida, with lower weights seen in Chesapeake Bay and higher weights in Florida [25,26] (Figure 3). The differences between our data and the Florida data were not significant, while the differences between our data and the Chesapeake Bay data were significant. This is potentially explained by the latitudinal differences between these three populations. In more temperate climates, as water temperatures drop in winter, blue crabs bury themselves in mud and enter a period of relative inactivity until waters warm in spring [9]. In Florida, where water temperatures are generally warm enough to support blue crabs over winter, individuals may be free to forage year-round, which may result in heavier masses at a given CW [26]. Seasonal biases in sampling may have also played a part in the differences between the stocks, since our sampling occurred primarily in the summer and the Chesapeake Bay survey occurred in the winter [9,27]. This also may explain the lack of a significant difference between our data and the Florida data, since our masses were exclusively collected in the summer when water temperatures are warm and crabs are actively gaining mass [9]. The crossover of the North Carolina function and the Florida function in males may simply be a result of low sample size on our part as well. We sourced our estimates from 345 males and 224 females, while the Florida and Chesapeake Bay stock assessments sourced their estimates from 3433 males and 1358 females, and ~5000 individuals of either sex, respectively [9,27]. It is possible the crossover between sizes is representative to some degree; male fiddler crab (*Uca uruguayensis*) carapace widths were found to range wider at more temperate latitudes than their tropical and subtropical conspecifics, but individual weights were not measured in this study [32].

Overall, we recommend that the Chesapeake Bay CW-to-weight values not be used in future North Carolina stock assessments because of the significant differences between the CW-to-weight estimates for blue crabs of both sexes. While no significant difference was found between the Florida estimates and the estimates produced by our data, we nonetheless recommend caution when using these estimates until further investigation can say whether the Florida estimates are truly representative of North Carolina CW-to-weight relationships.

The growth curves produced by our BFa model and our frequentist model were fairly similar in slope but had visibly different asymptotes set by their respective L_∞_ values. When compared to the other growth curves that we sourced, our L_∞_ estimates for our BFa and frequentist model fell between the other estimates [8,25,27,28,29] (Table 1 and Figure 5). Additionally, all L_∞_ estimates from other sources fell within the confidence interval of the BFa model [8,25,27,28,29] (Table 1). This suggests that both our L_∞_ estimates are biologically feasible representations of the North Carolina blue crab stock. Our estimates of K were the two highest K values when compared to the other estimates [8,25,27,28,29] (Table 1). Our frequentist estimate of K fell within the confidence interval of our BFa model, suggesting that these estimates were not a result of a modeling error.

We believe that these high K estimates are largely a product of our low rate of recapture for grown individuals, the timing of our recaptures, and the difference in methods between our study and the other studies to which we compared our results. While we recaptured and sacrificed 39 crabs, of these recaptures, 17 of them had not grown upon recapture. As such, we conducted our analysis using L1 and L2 data from the remaining 22 individuals that grew between initial capture and recapture. While our BFa analysis is built for data-limited scenarios [4], our estimates are likely still biased by our low sample size. Another source of bias is the timing of sampling and the timing of our recaptures. We sampled primarily from April to November of each year, with intensive sampling in May, June, July, and August. Given that all four of these months are late spring and summer months, our high K estimates and steep growth curve slopes may be due to our growth recaptures occurring primarily during the peak growing season for blue crabs. If more recaptures occurred in spring and fall when growth is slower, our K estimates would likely be more similar to the estimates from other sources. The other sources to which we compared our growth curves also used different methodology, had larger quantities of data, and often used data collected at different times of year and over long periods of time [8,25,27,28,29]. These methods are undoubtedly subject to their own sources of bias, but likely produce more accurate estimates of K in this case. Due to the similarity in AIC from most of the models we tested, we cannot definitively declare any model as the model of best fit. That said, the results of our model comparisons do provide information about the relationships between the parameters *p* and ϕ, the relationships between the groups and covariates tested, and the relationships between the parameters and the groups and covariates.

Due to the similarity in AIC from most of the CJS models we tested, we cannot definitively declare any model as the model of best fit. That said, the results of our model comparisons do provide information about the relationships between the parameters *p* and ϕ, the relationships between the groups and covariates tested, and the relationships between the parameters and the groups and covariates.

The observed negative relationship between the covariate CW and estimates of ϕ goes against the conventional contention that smaller blue crabs are subject to higher mortality than large blue crabs [9]. That said, it is important to note that the value of ϕ is a measure of apparent survival. Immigration and emigration are likely to have occurred during our study at all sites. As blue crabs increase in size and reach sexual maturity, they are subject to lower predation risk and will move more in search of food and mates [9]. Mature female blue crabs are known to migrate into high-salinity coastal habitats to spawn and do not return to the lower-salinity parts of the estuary for some time after spawning [33]. Johnson and Eggleston (2010) found in their CWT mark–recapture survey that loss rates are confounded by differences in CW and noted that emigration rates were lower in smaller vs. larger individuals [19]. MS and HS were also relatively open systems, which may explain their lower ϕ estimates compared to PC [19]. Further, individuals with a CW > 127 mm were likely subject to fishing mortality, especially as their CW approached the legal limit. While we took measures to avoid subjecting our individuals to fishing mortality (e.g., avoiding placing traps near other crab pots, not tagging individuals larger than the state size limit), some individuals may have potentially been subject to fishing mortality, either by traveling to an area with other crab pots or growing larger than the state size limit. This may also affect the models’ ability to properly model the relationship between CW and ϕ.

The inclusion of year as a group in some of our site-specific datasets is likely one of the main drivers behind the lower precision in both combined site treatments and PC treatments. ϕ values between years showed lower ϕ values in 2022, moderate ϕ values in 2023, and high ϕ values in 2024. This is likely due to differences in methodology over the three years of this study [34]. Without year as a group, our ϕ values ranged from 0.974 to 0.985; our lower acceptable threshold, based on Johnson and Eggleston 2010’s corrected ϕ estimate, was 0.94 [19]. At sites with 2022 included, all ϕ estimates for the year 2022 fall below Johnson and Eggleston 2010’s 0.94 value [19]. We expect that the calculated values at PC in 2024 are likely the most accurate estimates of M in the models we tested, as these ϕ estimates most closely align to the 2018 stock assessment models’ M range of 0.5–2.

The sex groupings’ effects on ϕ showed generally higher ϕ estimates in females than in males, but this relationship was not seen in all sex-grouped models. It is not uncommon for stock assessment models to calculate mortality independently for males and females, but studies that investigate sex-based differences in blue crab mortality often have difficulty isolating sex as the reason for differential mortality compared to size, morphology, or other contributing factors [9]. Additionally, the difference between male and female ϕ estimates may be a result of differential fishing mortality in individuals who are legal-sized [20]. Individuals of a given sex may also be emigrating from the area at higher or lower rates. With all these factors combined, we are unable to definitively say that sex is a major factor in determining apparent survival.

The complete dataset’s ϕ estimates when grouped by site were wide-ranging and imprecise. This is likely due to major differences in the timing of our sampling, accessible areas, number of sampling events, habitat, and other characteristics [34,35]. PC and MS were both accessible by wading at all times except the highest tides, but were generally sampled at low tide when individuals were concentrated into small areas. HS, on the other hand, was only accessible by boat, and had to be sampled at or around high tide to access the areas closest to salt marsh and other blue crab habitat. MS and PC were also more enclosed, while HS was a more open channel. PC was sampled the most out of the three sites; MS was only sampled during the 2024 season, and sampling at HS declined over the three years because of poor returns and technical difficulties with our boat. The use of site as a grouping variable instead of analyzing site separately can be useful, as ϕ estimates were less extreme when site is a grouping variable in the complete dataset. In this case, our complete data without site groupings has a considerably narrower range compared to the site-grouped data, suggesting that it may be oversimplifying its estimates of ϕ.

The covariate and group effects on model estimates of *p* were less clear: For CW, Year, Sex, and Site, the estimate of *p* did not follow a clear trend and often was seemingly dependent on the other covariates or groups. The only group that showed a clear effect on *p* was trap, which suggested that peeler pots resulted in a lower *p* estimate compared to minnow traps. This may be a result of differences in selectivity [35]. The trap grouping was only viable as a grouping at MS, which had relatively few captures. Because of this, we cannot definitively say that there is a clear relationship between *p* and trap used, and further studies may be necessary to understand what traps work best for mark–recapture surveys of blue crabs and other crustaceans.

The power of the groups and covariates to influence the model estimates of *p* and ϕ are clearly seen when comparing the respective estimates to each other (Figure 7). The ϕ estimates for PC with 2022 and both treatments of the complete dataset are considerably less precise than HS, MS, and PC without 2022 (Figure 7). This is likely due in part to the removal of non-viable groupings in the more precise treatments. The omission of 2022 in the PC dataset considerably improved its precision (Figure 7). The addition of site groupings to the complete dataset also widened the range of ϕ values calculated for the complete dataset and potentially interacted with the year groupings as well. This suggests that these grouping variables are informative and likely should be included in future mark–recapture studies seeking to estimate *p* and ϕ in blue crabs. Conversely, most of the values of *p* were fairly precise in all sites except MS, which used trap as a group (Figure 7). This suggests that most of the grouping variables we used were on the models’ estimation of *p*. This indicates that grouping capture and recapture data by gear type should be considered when estimating *p* if mark–recapture studies for blue crab use multiple gear types in the future.

Overall, we see the use of CWTs in mark–recapture surveys of blue crabs as a viable method for estimating growth and apparent survival, but considerable effort is necessary to ensure robust, accurate, and precise results. CWTs have been shown not to affect blue crab growth and long-term mortality [19,36]. Additionally, tag retention studies suggest that CWTs are retained long-term even after molting [19,36]. Future blue crab mark–recapture studies that use CWTs should carefully consider their capture methodology to ensure a large enough volume of crabs are captured and tagged to yield informative results.

## 5. Conclusions

The results of this study provide novel growth rate estimates using Frequentist and Bayesian Fabens methods. While our k estimates produced a steeper slope than is likely accurate for blue crab growth in general, it does demonstrate that the Bayesian Fabens approach could be useful at estimating growth in data-limited species. Our CW-to-width estimates for male and female blue crabs largely fell between the Florida and Chesapeake Bay estimates, suggesting that more localized estimates may be useful for future North Carolina stock assessments. Finally, while our CJS model did not determine a model of best fit for estimating apparent survival, we did identify covariates and groups that affected the estimates of the CJS model in meaningful ways. These results are useful for establishing which grouping variables and covariates would be informative if more mark–recapture data becomes available.

This study suggests that coded wire tags can be a viable option for mark–recapture surveys that measure growth of animals without permanent hard parts, including other crustacean and invertebrate species. That said, future mark–recapture studies using CWTs for crustaceans should expect to expend a considerable effort to yield meaningful results for growth and mortality estimates. To produce meaningful results, the authors recommend nearly closed sampling sites, frequent capture events, high saturation of traps, and short soak times to improve capture and recapture rates. Additionally, the Bayesian Fabens method did successfully converge with limited data, which suggests applications in data-limited species, including endangered species, species with low density, or species that are difficult to catch. Again, mark–recapture methodology should be carefully considered and year-round sampling should take place to ensure accurate and precise data is collected for the model.

## Figures and Tables

**Table 1:** Previously published estimates of von Bertalanffy growth parameters of blue crab, compared to this study’s estimates. Table of published estimates sourced from Miller et al. 2005 [25].

Source	Region	Data Source	Approach	K	L_∞_	t_0_
This Study	North Carolina	Mark–recapture survey	Bayesian Fabens approach	2.28	205.24	0
This Study	North Carolina	Mark–recapture survey	Frequentist Fabens approach	1.83	235.32	0
Rothschild et al. 1992 [27]	Chesapeake Bay	Fishery-independent survey	Modal analysis	1.08	176.0	0
Helser and Kahn 1999 [28]	Delaware	Fishery-independent survey	MULTIFAN	0.75	234.7	−0.10
Helser and Kahn 1999 [28]	Delaware	Fishery-independent survey	MULTIFAN	0.62	200.6	−0.15
Helser and Kahn 1999 [28]	Delaware	Fishery-independent survey	MULTIFAN	0.93	200.3	−0.15
Ju et al. 2001 [29]	Chesapeake Bay	Pond-reared individuals	Lipofuscin-based age and assumed max size	1.09	240.0	0.40
Eggleston and Johnson 2004 [8]	North Carolina	Fishery-independent survey	Modal analysis and assumed max size	0.47	216.9	0.02

## Data Availability

Data is available by request via contacting the Corresponding Author.

## Supplementary Materials

The following supporting information can be downloaded at: https://www.mdpi.com/article/10.3390/ani16152296/s1. Supplementary Table S1. Table of ϕ and p estimates from different model configurations and group combinations of the complete dataset, with accompanying AIC scores for each model. When the CW covariate is present, ϕ and p represent their respective means. Supplementary Table S2. Table of ϕ and p estimates from different model configurations and group combinations of the PC dataset including 2022, with accompanying AIC scores for each model. When the CW covariate is present, ϕ and p represent their respective means. Supplementary Table S3. Table of ϕ and p estimates from different model configurations and group combinations of the PC dataset excluding 2022, with accompanying AIC scores for each model. When the CW covariate is present, ϕ and p represent their respective means. Supplementary Table S4. Table of ϕ and p estimates from different model configurations and group combinations of the HS dataset, with accompanying AIC scores for each model. When the CW covariate is present, ϕ and p represent their respective means. Supplementary Table S5. Table of ϕ and p estimates from different model configurations and group combinations of the MS dataset, with accompanying AIC scores for each model. When the CW covariate is present, ϕ and p represent their respective means.
