# Supplementary material for: Estimating Survival, Growth, and Morphometric Relationships of Blue Crab (Callinectes sapidus) in North Carolina Using Mark–Recapture and Bayesian Approaches
Source: Animals (Basel). 2026 Jul 24;16(15):2296. doi: 10.3390/ani16152296 (PMC13463563; doi:10.3390/ani16152296)
Supplement: Supplementary file 1 [file animals-16-02296-s001.zip › animals-4420687-supplementary.pdf]

Supplementary Table S1. Table of ϕ and p estimates from different model configurations and group combinations of the complete dataset, with accompanying AIC scores for each model. When the CW covariate is present, ϕ and p represent their respective means.

P	Phi	Groups	Model Configuration	AICc
0.016151	0.977604	NA	Phi(~1)p(~1)	438.4515
0.018691	0.969618	NA	Phi(~CW)p(~1)	428.0218
0.013467	0.979167	NA	Phi(~1)p(~CW)	433.1029
0.004882	0.979888	Year2022	Phi(~1)p(~Year)	433.1592
0.011185	0.979888	Year2023	Phi(~1)p(~Year)	433.1592
0.021537	0.979888	Year2024	Phi(~1)p(~Year)	433.1592
0.013566	0.981362	SexF	Phi(~Sex)p(~CW)	434.8873
0.013566	0.977004	SexM	Phi(~Sex)p(~CW)	434.8873
0.018651	0.969655	NA	Phi(~CW)p(~CW)	430.0495
0.014486	0.928185	Year2022	Phi(~Year)p(~CW)	428.6021
0.014486	0.968279	Year2023	Phi(~Year)p(~CW)	428.6021
0.014486	0.989475	Year2024	Phi(~Year)p(~CW)	428.6021
0.004747	0.98366	SexF.Year2022	Phi(~Sex)p(~Year)	434.5015
0.004747	0.976138	SexM.Year2022	Phi(~Sex)p(~Year)	434.5015
0.011	0.98366	SexF.Year2023	Phi(~Sex)p(~Year)	434.5015
0.011	0.976138	SexM.Year2023	Phi(~Sex)p(~Year)	434.5015
0.022259	0.98366	SexF.Year2024	Phi(~Sex)p(~Year)	434.5015
0.022259	0.976138	SexM.Year2024	Phi(~Sex)p(~Year)	434.5015
0.007656	0.972792	Year2022	Phi(~CW)p(~Year)	428.5998
0.015177	0.972792	Year2023	Phi(~CW)p(~Year)	428.5998
0.021352	0.972792	Year2024	Phi(~CW)p(~Year)	428.5998
0.014177	0.922965	Year2022	Phi(~Year)p(~Year)	432.542
0.018152	0.961806	Year2023	Phi(~Year)p(~Year)	432.542

0.016223	0.990374	Year2024	Phi(~Year)p(~Year)	432.542
0.005627	0.980332	Year2022	Phi(~1)p(~CW + Year)	433.3196
0.011678	0.980332	Year2023	Phi(~1)p(~CW + Year)	433.3196
0.017693	0.980332	Year2024	Phi(~1)p(~CW + Year)	433.3196
0.007849	0.9711	Year2022	Phi(~CW)p(~CW + Year)	430.2158
0.016274	0.9711	Year2023	Phi(~CW)p(~CW + Year)	430.2158
0.024466	0.9711	Year2024	Phi(~CW)p(~CW + Year)	430.2158
0.010509	0.957967	Year2022	Phi(~CW + Year)p(~CW + Year)	433.7998
0.0193	0.963647	Year2023	Phi(~CW + Year)p(~CW + Year)	433.7998
0.019913	0.979011	Year2024	Phi(~CW + Year)p(~CW + Year)	433.7998
0.017076	0.937271	Year2022	Phi(~CW + Year)p(~CW)	430.0031
0.017076	0.965293	Year2023	Phi(~CW + Year)p(~CW)	430.0031
0.017076	0.983382	Year2024	Phi(~CW + Year)p(~CW)	430.0031
0.010898	0.956071	Year2022	Phi(~CW + Year)p(~Year)	431.7528
0.01932	0.963372	Year2023	Phi(~CW + Year)p(~Year)	431.7528
0.019158	0.980012	Year2024	Phi(~CW + Year)p(~Year)	431.7528
0.01342	0.950912	SexF.Year2022	Phi(~Sex + CW + Year)p(~Year)	433.4517
0.01342	0.9313	SexM.Year2022	Phi(~Sex + CW + Year)p(~Year)	433.4517
0.01695	0.973248	SexF.Year2023	Phi(~Sex + CW + Year)p(~Year)	433.4517
0.01695	0.962204	SexM.Year2023	Phi(~Sex + CW + Year)p(~Year)	433.4517
0.019721	0.983786	SexF.Year2024	Phi(~Sex + CW + Year)p(~Year)	433.4517
0.019721	0.976989	SexM.Year2024	Phi(~Sex + CW + Year)p(~Year)	433.4517
0.016157	0.941248	SexF.Year2022	Phi(~Sex + CW + Year)p(~CW + Year)	435.4811

0.016157	0.910619	SexM.Year2022	Phi(~Sex + CW + Year)p(~CW + Year)	435.4811
0.016457	0.974847	SexF.Year2023	Phi(~Sex + CW + Year)p(~CW + Year)	435.4811
0.016457	0.961008	SexM.Year2023	Phi(~Sex + CW + Year)p(~CW + Year)	435.4811
0.017504	0.987296	SexF.Year2024	Phi(~Sex + CW + Year)p(~CW + Year)	435.4811
0.017504	0.980167	SexM.Year2024	Phi(~Sex + CW + Year)p(~CW + Year)	435.4811
0.018928	0.891786	SiteHS	Phi(~Site)p(~1)	432.5862
0.018928	0.955396	SiteMS	Phi(~Site)p(~1)	432.5862
0.018928	0.980895	SitePC	Phi(~Site)p(~1)	432.5862
0.018977	0.891647	SiteHS	Phi(~Site)p(~Site)	436.456
0.025378	0.945359	SiteMS	Phi(~Site)p(~Site)	436.456
0.018209	0.981507	SitePC	Phi(~Site)p(~Site)	436.456
0.02305	0.893661	SiteHS	Phi(~Site)p(~CW + Site)	433.9077
0.020773	0.946146	SiteMS	Phi(~Site)p(~CW + Site)	433.9077
0.015135	0.982735	SitePC	Phi(~Site)p(~CW + Site)	433.9077
0.017884	0.926302	SiteHS	Phi(~CW + Site)p(~Site)	431.1315
0.02127	0.942618	SiteMS	Phi(~CW + Site)p(~Site)	431.1315
0.020468	0.97456	SitePC	Phi(~CW + Site)p(~Site)	431.1315
0.017661	0.927223	SiteHS	Phi(~CW + Site)p(~CW + Site)	433.1817
0.021482	0.942507	SiteMS	Phi(~CW + Site)p(~CW + Site)	433.1817
0.020786	0.974276	SitePC	Phi(~CW + Site)p(~CW + Site)	433.1817
0.018993	0.887409	SexF.SiteHS	Phi(~Sex + Site)p(~Site)	438.4635
0.018993	0.895722	SexM.SiteHS	Phi(~Sex + Site)p(~Site)	438.4635
0.026235	0.939888	SexF.SiteMS	Phi(~Sex + Site)p(~Site)	438.4635

0.026235	0.944568	SexM.SiteMS	Phi(~Sex + Site)p(~Site)	438.4635
0.018098	0.980819	SexF.SitePC	Phi(~Sex + Site)p(~Site)	438.4635
0.018098	0.982372	SexM.SitePC	Phi(~Sex + Site)p(~Site)	438.4635
0.016036	0.892614	SexF.SiteHS	Phi(~Site)p(~Sex + Site)	437.8015
0.021447	0.892614	SexM.SiteHS	Phi(~Site)p(~Sex + Site)	437.8015
0.019811	0.94628	SexF.SiteMS	Phi(~Site)p(~Sex + Site)	437.8015
0.026461	0.94628	SexM.SiteMS	Phi(~Site)p(~Sex + Site)	437.8015
0.015338	0.981466	SexF.SitePC	Phi(~Site)p(~Sex + Site)	437.8015
0.020519	0.981466	SexM.SitePC	Phi(~Site)p(~Sex + Site)	437.8015
0.010753	0.878549	Year2022.SiteHS	Phi(~Site)p(~Year + Site)	431.8497
0.030799	0.878549	Year2023.SiteHS	Phi(~Site)p(~Year + Site)	431.8497
0.050639	0.878549	Year2024.SiteHS	Phi(~Site)p(~Year + Site)	431.8497
0.025378	0.945359	Year2024.SiteMS	Phi(~Site)p(~Year + Site)	431.8497
0.005055	0.983623	Year2022.SitePC	Phi(~Site)p(~Year + Site)	431.8497
0.014635	0.983623	Year2023.SitePC	Phi(~Site)p(~Year + Site)	431.8497
0.024324	0.983623	Year2024.SitePC	Phi(~Site)p(~Year + Site)	431.8497
0.012381	0.86585	Year2022.SiteHS	Phi(~Year + Site)p(~Site)	428.5225
0.012381	0.941301	Year2023.SiteHS	Phi(~Year + Site)p(~Site)	428.5225
0.012381	0.987695	Year2024.SiteHS	Phi(~Year + Site)p(~Site)	428.5225
0.025378	0.945359	Year2024.SiteMS	Phi(~Year + Site)p(~Site)	428.5225
0.018612	0.926493	Year2022.SitePC	Phi(~Year + Site)p(~Site)	428.5225
0.018612	0.969055	Year2023.SitePC	Phi(~Year + Site)p(~Site)	428.5225
0.018612	0.993661	Year2024.SitePC	Phi(~Year + Site)p(~Site)	428.5225
0.010023	0.869911	Year2022.SiteHS	Phi(~Year + Site)p(~Year + Site)	432.012
0.024166	0.899825	Year2023.SiteHS	Phi(~Year + Site)p(~Year + Site)	432.012

0.014981	0.985961	Year2024.SiteHS	Phi(~Year + Site)p(~Year + Site)	432.012
0.025378	0.945359	Year2024.SiteMS	Phi(~Year + Site)p(~Year + Site)	432.012
0.011645	0.949218	Year2022.SitePC	Phi(~Year + Site)p(~Year + Site)	432.012
0.028013	0.961698	Year2023.SitePC	Phi(~Year + Site)p(~Year + Site)	432.012
0.017392	0.994932	Year2024.SitePC	Phi(~Year + Site)p(~Year + Site)	432.012
0.010753	0.878549	Year2022.SiteHS	Phi(~Site)p(~Year + Site)	431.8497
0.030799	0.878549	Year2023.SiteHS	Phi(~Site)p(~Year + Site)	431.8497
0.050639	0.878549	Year2024.SiteHS	Phi(~Site)p(~Year + Site)	431.8497
0.025378	0.945359	Year2024.SiteMS	Phi(~Site)p(~Year + Site)	431.8497
0.005055	0.983623	Year2022.SitePC	Phi(~Site)p(~Year + Site)	431.8497
0.014635	0.983623	Year2023.SitePC	Phi(~Site)p(~Year + Site)	431.8497
0.024324	0.983623	Year2024.SitePC	Phi(~Site)p(~Year + Site)	431.8497
0.019714	0.894543	SexF.Year2022.SiteHS	Phi(~Site)p(~Sex + CW + Site)	435.4407
0.025322	0.894543	SexM.Year2022.SiteHS	Phi(~Site)p(~Sex + CW + Site)	435.4407
0.019714	0.894543	SexF.Year2023.SiteHS	Phi(~Site)p(~Sex + CW + Site)	435.4407
0.025322	0.894543	SexM.Year2023.SiteHS	Phi(~Site)p(~Sex + CW + Site)	435.4407
0.025322	0.894543	SexM.Year2024.SiteHS	Phi(~Site)p(~Sex + CW + Site)	435.4407
0.016856	0.94696	SexF.Year2024.SiteMS	Phi(~Site)p(~Sex + CW + Site)	435.4407
0.021669	0.94696	SexM.Year2024.SiteMS	Phi(~Site)p(~Sex + CW + Site)	435.4407
0.013072	0.982667	SexF.Year2022.SitePC	Phi(~Site)p(~Sex + CW + Site)	435.4407
0.016823	0.982667	SexM.Year2022.SitePC	Phi(~Site)p(~Sex + CW + Site)	435.4407
0.013072	0.982667	SexF.Year2023.SitePC	Phi(~Site)p(~Sex + CW + Site)	435.4407
0.016823	0.982667	SexM.Year2023.SitePC	Phi(~Site)p(~Sex + CW + Site)	435.4407

0.013072	0.982667	SexF.Year2024.SitePC	Phi(~Site)p(~Sex + CW + Site)	435.4407
0.016823	0.982667	SexM.Year2024.SitePC	Phi(~Site)p(~Sex + CW + Site)	435.4407
0.017924	0.920543	SexF.Year2022.SiteHS	Phi(~Sex + CW + Site)p(~Site)	433.0762
0.017924	0.930602	SexM.Year2022.SiteHS	Phi(~Sex + CW + Site)p(~Site)	433.0762
0.017924	0.920543	SexF.Year2023.SiteHS	Phi(~Sex + CW + Site)p(~Site)	433.0762
0.017924	0.930602	SexM.Year2023.SiteHS	Phi(~Sex + CW + Site)p(~Site)	433.0762
0.017924	0.930602	SexM.Year2024.SiteHS	Phi(~Sex + CW + Site)p(~Site)	433.0762
0.022327	0.932107	SexF.Year2024.SiteMS	Phi(~Sex + CW + Site)p(~Site)	433.0762
0.022327	0.940796	SexM.Year2024.SiteMS	Phi(~Sex + CW + Site)p(~Site)	433.0762
0.020261	0.972901	SexF.Year2022.SitePC	Phi(~Sex + CW + Site)p(~Site)	433.0762
0.020261	0.976501	SexM.Year2022.SitePC	Phi(~Sex + CW + Site)p(~Site)	433.0762
0.020261	0.972901	SexF.Year2023.SitePC	Phi(~Sex + CW + Site)p(~Site)	433.0762
0.020261	0.976501	SexM.Year2023.SitePC	Phi(~Sex + CW + Site)p(~Site)	433.0762
0.020261	0.972901	SexF.Year2024.SitePC	Phi(~Sex + CW + Site)p(~Site)	433.0762
0.020261	0.976501	SexM.Year2024.SitePC	Phi(~Sex + CW + Site)p(~Site)	433.0762
0.014357	0.933287	SexF.Year2022.SiteHS	Phi(~Sex + CW + Site)p(~Sex + CW + Site)	436.7591
0.020159	0.922153	SexM.Year2022.SiteHS	Phi(~Sex + CW + Site)p(~Sex + CW + Site)	436.7591
0.014357	0.933287	SexF.Year2023.SiteHS	Phi(~Sex + CW + Site)p(~Sex + CW + Site)	436.7591
0.020159	0.922153	SexM.Year2023.SiteHS	Phi(~Sex + CW + Site)p(~Sex + CW + Site)	436.7591
0.020159	0.922153	SexM.Year2024.SiteHS	Phi(~Sex + CW + Site)p(~Sex + CW + Site)	436.7591
0.015726	0.952307	SexF.Year2024.SiteMS	Phi(~Sex + CW + Site)p(~Sex + CW + Site)	436.7591

0.02207	0.944158	SexM.Year2024.SiteMS	Phi(~Sex + CW + Site)p(~Sex + CW + Site)	436.7591
0.016963	0.976457	SexF.Year2022.SitePC	Phi(~Sex + CW + Site)p(~Sex + CW + Site)	436.7591
0.023794	0.972314	SexM.Year2022.SitePC	Phi(~Sex + CW + Site)p(~Sex + CW + Site)	436.7591
0.016963	0.976457	SexF.Year2023.SitePC	Phi(~Sex + CW + Site)p(~Sex + CW + Site)	436.7591
0.023794	0.972314	SexM.Year2023.SitePC	Phi(~Sex + CW + Site)p(~Sex + CW + Site)	436.7591
0.016963	0.976457	SexF.Year2024.SitePC	Phi(~Sex + CW + Site)p(~Sex + CW + Site)	436.7591
0.023794	0.972314	SexM.Year2024.SitePC	Phi(~Sex + CW + Site)p(~Sex + CW + Site)	436.7591

Supplementary Table S2. Table of ϕ and p estimates from different model configurations and group combinations of the PC dataset including 2022, with accompanying AIC scores for each model. When the CW covariate is present, ϕ and p represent their respective means.

**P**	**Phi**	**Groups**	**Model Configuration**	**AICc**
0.013797	0.98214	Year2022	Phi(~1)p(~Year)	304.3806
0.024824	0.98214	Year2023	Phi(~1)p(~Year)	304.3806
0.037815	0.98214	Year2024	Phi(~1)p(~Year)	304.3806
0.019693	0.978719	Year2022	Phi(~CW)p(~Year)	303.3249
0.028396	0.978719	Year2023	Phi(~CW)p(~Year)	303.3249
0.036977	0.978719	Year2024	Phi(~CW)p(~Year)	303.3249
0.047383	0.921666	Year2022	Phi(~Year)p(~Year)	302.7857
0.048532	0.961333	Year2023	Phi(~Year)p(~Year)	302.7857
0.025559	0.995806	Year2024	Phi(~Year)p(~Year)	302.7857
0.041837	0.938216	Year2022	Phi(~CW + Year)p(~Year)	304.7455
0.04839	0.9618	Year2023	Phi(~CW + Year)p(~Year)	304.7455
0.027601	0.992927	Year2024	Phi(~CW + Year)p(~Year)	304.7455
0.020106	0.978778	SexF.Year2022	Phi(~CW)p(~Sex + Year)	305.3722
0.01899	0.978778	SexM.Year2022	Phi(~CW)p(~Sex + Year)	305.3722
0.029163	0.978778	SexF.Year2023	Phi(~CW)p(~Sex + Year)	305.3722
0.027558	0.978778	SexM.Year2023	Phi(~CW)p(~Sex + Year)	305.3722
0.038305	0.978778	SexF.Year2024	Phi(~CW)p(~Sex + Year)	305.3722
0.036215	0.978778	SexM.Year2024	Phi(~CW)p(~Sex + Year)	305.3722
0.050136	0.921519	SexF.Year2022	Phi(~Year)p(~Sex + Year)	304.7296
0.043662	0.921519	SexM.Year2022	Phi(~Year)p(~Sex + Year)	304.7296
0.052252	0.961318	SexF.Year2023	Phi(~Year)p(~Sex + Year)	304.7296
0.045518	0.961318	SexM.Year2023	Phi(~Year)p(~Sex + Year)	304.7296
0.027831	0.996025	SexF.Year2024	Phi(~Year)p(~Sex + Year)	304.7296

0.024164	0.996025	SexM.Year2024	Phi(~Year)p(~Sex + Year)	304.7296
0.044837	0.936104	SexF.Year2022	Phi(~CW + Year)p(~Sex + Year)	306.7332
0.039635	0.936104	SexM.Year2022	Phi(~CW + Year)p(~Sex + Year)	306.7332
0.05176	0.961664	SexF.Year2023	Phi(~CW + Year)p(~Sex + Year)	306.7332
0.045793	0.961664	SexM.Year2023	Phi(~CW + Year)p(~Sex + Year)	306.7332
0.029507	0.99349	SexF.Year2024	Phi(~CW + Year)p(~Sex + Year)	306.7332
0.026035	0.99349	SexM.Year2024	Phi(~CW + Year)p(~Sex + Year)	306.7332
0.01932	0.97758	Year2022	Phi(~CW)p(~CW + Year)	305.08
0.030629	0.97758	Year2023	Phi(~CW)p(~CW + Year)	305.08
0.041422	0.97758	Year2024	Phi(~CW)p(~CW + Year)	305.08
0.062652	0.916301	Year2022	Phi(~Year)p(~CW + Year)	303.9489
0.04782	0.961671	Year2023	Phi(~Year)p(~CW + Year)	303.9489
0.021629	0.996822	Year2024	Phi(~Year)p(~CW + Year)	303.9489
0.080889	0.883591	Year2022	Phi(~CW + Year)p(~CW + Year)	305.6043
0.046957	0.962827	Year2023	Phi(~CW + Year)p(~CW + Year)	305.6043
0.018407	0.999399	Year2024	Phi(~CW + Year)p(~CW + Year)	305.6043
0.019843	0.977621	SexF.Year2022	Phi(~CW)p(~Sex + CW + Year)	307.1227
0.018383	0.977621	SexM.Year2022	Phi(~CW)p(~Sex + CW + Year)	307.1227
0.031827	0.977621	SexF.Year2023	Phi(~CW)p(~Sex + CW + Year)	307.1227
0.029512	0.977621	SexM.Year2023	Phi(~CW)p(~Sex + CW + Year)	307.1227
0.043568	0.977621	SexF.Year2024	Phi(~CW)p(~Sex + CW + Year)	307.1227
0.040435	0.977621	SexM.Year2024	Phi(~CW)p(~Sex + CW + Year)	307.1227
0.018805	0.981804	SexF.Year2022	Phi(~Sex + CW)p(~Year)	305.0413
0.018805	0.97555	SexM.Year2022	Phi(~Sex + CW)p(~Year)	305.0413
0.028314	0.981804	SexF.Year2023	Phi(~Sex + CW)p(~Year)	305.0413
0.028314	0.97555	SexM.Year2023	Phi(~Sex + CW)p(~Year)	305.0413

0.037993	0.981804	SexF.Year2024	Phi(~Sex + CW)p(~Year)	305.0413
0.037993	0.97555	SexM.Year2024	Phi(~Sex + CW)p(~Year)	305.0413
0.013247	0.985428	SexF.Year2022	Phi(~Sex)p(~Year)	305.6735
0.013247	0.977923	SexM.Year2022	Phi(~Sex)p(~Year)	305.6735
0.025093	0.985428	SexF.Year2023	Phi(~Sex)p(~Year)	305.6735
0.025093	0.977923	SexM.Year2023	Phi(~Sex)p(~Year)	305.6735
0.039327	0.985428	SexF.Year2024	Phi(~Sex)p(~Year)	305.6735
0.039327	0.977923	SexM.Year2024	Phi(~Sex)p(~Year)	305.6735
0.018387	0.98076	SexF.Year2022	Phi(~Sex + CW)p(~CW + Year)	306.8225
0.018387	0.974444	SexM.Year2022	Phi(~Sex + CW)p(~CW + Year)	306.8225
0.030439	0.98076	SexF.Year2023	Phi(~Sex + CW)p(~CW + Year)	306.8225
0.030439	0.974444	SexM.Year2023	Phi(~Sex + CW)p(~CW + Year)	306.8225
0.042381	0.98076	SexF.Year2024	Phi(~Sex + CW)p(~CW + Year)	306.8225
0.042381	0.974444	SexM.Year2024	Phi(~Sex + CW)p(~CW + Year)	306.8225

Supplementary Table S3. Table of ϕ and p estimates from different model configurations and group combinations of the PC dataset excluding 2022, with accompanying AIC scores for each model. When the CW covariate is present, ϕ and p represent their respective means.

P	Phi	Groups	Model Configuration	AICc
0.0241352	0.9845441	Year2023	Phi(~1)p(~Year)	279.28194
0.0355617	0.9845441	Year2024	Phi(~1)p(~Year)	279.28194
0.0291611	0.9837514	Year2023	Phi(~CW)p(~Year)	278.5151
0.0355441	0.9837514	Year2024	Phi(~CW)p(~Year)	278.5151
0.0517935	0.9618859	Year2023	Phi(~Year)p(~Year)	277.51519
0.0255591	0.9958055	Year2024	Phi(~Year)p(~Year)	277.51519
0.0487304	0.968897	Year2023	Phi(~CW + Year)p(~Year)	278.91869
0.0293588	0.991224	Year2024	Phi(~CW + Year)p(~Year)	278.91869
0.0283945	0.9837104	SexF.Year2023	Phi(~CW)p(~Sex + Year)	280.58529
0.029951	0.9837104	SexM.Year2023	Phi(~CW)p(~Sex + Year)	280.58529
0.0343618	0.9837104	SexF.Year2024	Phi(~CW)p(~Sex + Year)	280.58529
0.0362332	0.9837104	SexM.Year2024	Phi(~CW)p(~Sex + Year)	280.58529
0.0529146	0.9618883	SexF.Year2023	Phi(~Year)p(~Sex + Year)	279.59339
0.0509453	0.9618883	SexM.Year2023	Phi(~Year)p(~Sex + Year)	279.59339
0.0261751	0.9958691	SexF.Year2024	Phi(~Year)p(~Sex + Year)	279.59339
0.0251745	0.9958691	SexM.Year2024	Phi(~Year)p(~Sex + Year)	279.59339
0.0484588	0.9689223	SexF.Year2023	Phi(~CW + Year)p(~Sex + Year)	281.02407
0.0489152	0.9689223	SexM.Year2023	Phi(~CW + Year)p(~Sex + Year)	281.02407
0.0291949	0.9911986	SexF.Year2024	Phi(~CW + Year)p(~Sex + Year)	281.02407
0.0294755	0.9911986	SexM.Year2024	Phi(~CW + Year)p(~Sex + Year)	281.02407
0.0292351	0.9837105	Year2023	Phi(~CW)p(~CW + Year)	280.60104
0.0357281	0.9837105	Year2024	Phi(~CW)p(~CW + Year)	280.60104
0.0560165	0.9624284	Year2023	Phi(~Year)p(~CW + Year)	278.07471

0.02274	0.9971026	Year2024	Phi(~Year)p(~CW + Year)	278.07471
0.0572126	0.9607755	Year2023	Phi(~CW + Year)p(~CW + Year)	280.12603
0.0216174	0.9982695	Year2024	Phi(~CW + Year)p(~CW + Year)	280.12603
0.0284631	0.9836799	SexF.Year2023	Phi(~CW)p(~Sex + CW + Year)	282.69018
0.0299944	0.9836799	SexM.Year2023	Phi(~CW)p(~Sex + CW + Year)	282.69018
0.0345198	0.9836799	SexF.Year2024	Phi(~CW)p(~Sex + CW + Year)	282.69018
0.0363648	0.9836799	SexM.Year2024	Phi(~CW)p(~Sex + CW + Year)	282.69018
0.0288737	0.9860517	SexF.Year2023	Phi(~Sex + CW)p(~Year)	280.37441
0.0288737	0.9813883	SexM.Year2023	Phi(~Sex + CW)p(~Year)	280.37441
0.0362909	0.9860517	SexF.Year2024	Phi(~Sex + CW)p(~Year)	280.37441
0.0362909	0.9813883	SexM.Year2024	Phi(~Sex + CW)p(~Year)	280.37441
0.0242577	0.9883036	SexF.Year2023	Phi(~Sex)p(~Year)	280.43447
0.0242577	0.980204	SexM.Year2023	Phi(~Sex)p(~Year)	280.43447
0.0369486	0.9883036	SexF.Year2024	Phi(~Sex)p(~Year)	280.43447
0.0369486	0.980204	SexM.Year2024	Phi(~Sex)p(~Year)	280.43447
0.0287869	0.9861354	SexF.Year2023	Phi(~Sex + CW)p(~CW + Year)	282.47819
0.0287869	0.9813953	SexM.Year2023	Phi(~Sex + CW)p(~CW + Year)	282.47819
0.0360966	0.9861354	SexF.Year2024	Phi(~Sex + CW)p(~CW + Year)	282.47819
0.0360966	0.9813953	SexM.Year2024	Phi(~Sex + CW)p(~CW + Year)	282.47819

Supplementary Table S4. Table of ϕ and p estimates from different model configurations and group combinations of the HS dataset, with accompanying AIC scores for each model. When the CW covariate is present, ϕ and p represent their respective means.

P	Phi	Groups	Model Configuration	AICc
0.292202	0.770076	NA	Phi(~1)p(~1)	22.39119
0.291704	0.769526	SexF	Phi(~Sex)p(~1)	24.48939
0.291704	0.770925	SexM	Phi(~Sex)p(~1)	24.48939
0.284137	0.772122	NA	Phi(~CW)p(~1)	24.44948
0.307044	0.769068	SexF	Phi(~1)p(~Sex)	24.48679
0.285101	0.769068	SexM	Phi(~1)p(~Sex)	24.48679
0.291102	0.770261	NA	Phi(~1)p(~CW)	24.46756
0.449101	0.737713	SexF	Phi(~Sex)p(~Sex)	26.58999
0.220075	0.792255	SexM	Phi(~Sex)p(~Sex)	26.58999
0.31236	0.770746	SexF	Phi(~CW)p(~Sex)	26.57084
0.267414	0.770746	SexM	Phi(~CW)p(~Sex)	26.57084
0.292706	0.7713	SexF	Phi(~Sex)p(~CW)	26.59966
0.292706	0.768309	SexM	Phi(~Sex)p(~CW)	26.59966

Supplementary Table S5. Table of ϕ and p estimates from different model configurations and group combinations of the MS dataset, with accompanying AIC scores for each model. When the CW covariate is present, ϕ and p represent their respective means.

**P**	**Phi**	**Groups**	**Model Configuration**	**AICc**
0.049373	0.944372	NA	Phi(~1)p(~1)	63.41064
0.0476	0.947096	NA	Phi(~CW)p(~1)	65.17717
0.048855	0.944873	NA	Phi(~1)p(~CW)	65.37493
0.100975	0.942829	TrapMT	Phi(~1)p(~Trap)	62.91939
0.025504	0.942829	TrapPP	Phi(~1)p(~Trap)	62.91939
0.048123	0.972806	NA	Phi(~CW)p(~CW)	65.95145
0.098263	0.946085	TrapMT	Phi(~CW)p(~Trap)	64.60879
0.024082	0.946085	TrapPP	Phi(~CW)p(~Trap)	64.60879
0.097114	0.944402	TrapMT	Phi(~1)p(~CW + Trap)	64.99653
0.024936	0.944402	TrapPP	Phi(~1)p(~CW + Trap)	64.99653
0.246041	0.941986	TrapMT	Phi(~CW)p(~CW + Trap)	64.74012
0.035424	0.941986	TrapPP	Phi(~CW)p(~CW + Trap)	64.74012
